# Survival outcomes following treatment delays among patients with early-stage female cancers: a nationwide study

**DOI:** 10.1186/s12967-022-03719-7

**Published:** 2022-12-03

**Authors:** Yu Min, Zheran Liu, Rendong Huang, Ruidan Li, Jing Jin, Zhigong Wei, Ling He, Yiyan Pei, Ning Li, Yongllin Su, Xiaolin Hu, Xingchen Peng

**Affiliations:** 1 Department of Biotherapy and National Clinical Research Center for Geriatrics, Cancer Center, West China Hospital, Sichuan University, Chengdu, 610041 Sichuan China; 2 School of Nursing, Hangzhou Medical College, Hangzhou, Zhejiang China; 3 Affiliated Hospital of Nanjing University of Chinese Medicine, Nanjing, Jiangsu China; 4 Department of Rehabilitation, Cancer Center, West China Hospital, Sichuan University, Chengdu, 610041 Sichuan China; 5 West China School of Nursing, West China Hospital, Sichuan University, Chengdu, 610041 Sichuan China

**Keywords:** Treatment delay intervals, Risk factors, Female cancer, SEER, Survival, Marital status

## Abstract

**Background:**

The coronavirus disease 2019 (COVID-19) severely hindered the timely receipt of health care for patients with cancer, especially female patients. Depression and anxiety were more pronounced in female patients than their male counterparts with cancer during treatment wait-time intervals. Herein, investigating the impact of treatment delays on the survival outcomes of female patients with early-stage cancers can enhance the rational and precise clinical decisions of physicians.

**Methods:**

We analyzed five types of cancers in women from the Surveillance, Epidemiology, and End Results (SEER) program between Jan 2010 and Dec 2015. Univariate and multivariate Cox regression analyses were used to determine the impacts of treatment delays on the overall survival (OS) and cancer-specific survival (CSS) of the patients.

**Results:**

A total of 241,661 females with early-stage cancer were analyzed (12,617 cases of non-small cell lung cancer (NSCLC), 166,051 cases of infiltrating breast cancer, 31,096 cases of differentiated thyroid cancer, 23,550 cases of colorectal cancer, and 8347 cases of cervical cancer). Worse OS rates were observed in patients with treatment delays ≥ 3 months in stage I NSCLC (_adjusted_Hazard ratio (HR) = 1.11, 95% Confidence Interval (CI): 1.01–1.23, p = 0.044) and stage I infiltrating breast cancer (_adjusted_HR = 1.23, 95% CI 1.11–1.37, p < 0.001). When the treatment delay intervals were analyzed as continuous variables, similar results were observed in patients with stage I NSCLC (_adjusted_HR = 1.04, 95% CI 1.01–1.06, p = 0.010) and in those with stage I breast cancer (_adjusted_HR = 1.03, 95% CI 1.00–1.06, p = 0.029). However, treatment delays did not reduce the OS of patients with differentiated thyroid cancer, cervical cancer, or colorectal cancer in the early-stage. Only intermediate treatment delays impaired the CSS of patients with cervical cancer in stage I (_adjusted_HR = 1.31, 95% CI 1.02–1.68, p = 0.032).

**Conclusion:**

After adjusting for confounders, the prolonged time from diagnosis to the initiation of treatment (< 6 months) showed limited negative effects on the survival of most of the patients with early-stage female cancers. Whether our findings serve as evidence supporting the treatment deferral decisions of clinicians for patients with different cancers in resource-limited situations needs further validation.

**Supplementary Information:**

The online version contains supplementary material available at 10.1186/s12967-022-03719-7.

## Introduction

Cancer has become a critical public health concern worldwide, and the social burden of cancer will continue to increase along with the aging population [1]. The coronavirus disease 2019 (COVID-19) pandemic caused major disruptions in healthcare delivery worldwide, putting patients with cancer at higher risk for negative outcomes from prolonged delays in their diagnosis and treatment [2, 3]. Delays in the treatment of patients with localized cancers after their initial diagnoses increased their likelihood of developing locally advanced and even metastatic disease. Continuous updates of evidence derived from systematic reviews and meta-analyses show that discrepancies in the impacts of prolonged wait times from diagnosis to treatment on clinical outcomes were observed in patients with various types of cancers [4–6].

Before the COVID-19 pandemic, several studies reported that delays in treatment increased the probability of all-cause mortality in patients with different types of cancers, including but not limited to, endometrial cancer [7], liver cancer [8], breast cancer [9], and oral cavity squamous cell carcinoma [10]. However, accumulative evidence showed that a prolonged delay in treatment was not associated with an increased risk of adverse outcomes in patients with some cancers, such as a 15-day delay for patients with acute myeloid leukemia [11] and 2 month delays for patients with curable gastric cancer [12] and advanced pancreatic cancer [13]. Therefore, the length of time that is acceptable (i.e., safe from disease progression) within the interval between the diagnosis and treatment of cancer is debatable. More population-based studies from different regions could help healthcare providers rethink the impact of prolonged time from diagnosis to treatment initiation on the progression of cancers and patients’ survival.

Current evidence shows that the incidence of cancer among women has increased (0.2% per year) annually in recent years, whereas cancer rates among men have generally decreased since the early 1990s. This change has resulted in the narrowing of the sex gap in incidence, with the male-to-female incidence ratio declining from 1.39 in 1995 to 1.14 in 2018 [14]. Recent data show that depression and anxiety were more pronounced among female patients during the COVID-19 pandemic [15, 16]. Unfortunately, female patients were more likely than their male counterparts to experience prolonged wait times between symptom presentation and diagnosis, as well as the initiation of treatment [17–19]. Frequent sex disparities with a predominance of women have been reported in some cancers, such as differentiated thyroid carcinomas (DTC) [20]. Yet few studies have investigated this topic in samples with female-specific cancers, such as cervical cancer [21, 22]. Thus, a deep understanding of the association between treatment delay intervals and survival outcomes could provide useful and rational guidance for clinical practice.

Thus, we aim to investigate the impact of treatment delays on the long-term overall survival (OS) and cancer-specific survival (CSS) of patients with one of the five female cancers with the highest incidence rates, using a nationwide population-based dataset. We also analyze the 10-year predictions of OS patterns in patients with one of the five types of cancers and different treatment delay intervals.

## Materials and methods

### Data source

In this retrospective observational study, we evaluated the five most common cancers diagnosed in women (breast cancer, lung cancer, colorectal cancer, cervical cancer, and thyroid cancer) [1]. Patients’ monthly records from diagnosis to treatment were added to the list of data, in accordance with the latest version of the Surveillance, Epidemiology, and End Results (SEER) program. The data were derived from the SEER program, which covers 17 states of the United States of America and approximately 28% of its population consisting of various races and ethnicities. The period of data collection was from 2010 to 2015. The present study followed the checklist of the Strengthening the Reporting of Observational Studies in Epidemiology (STROBE) statement [23].

### Patient selection

**Fig. 1:**
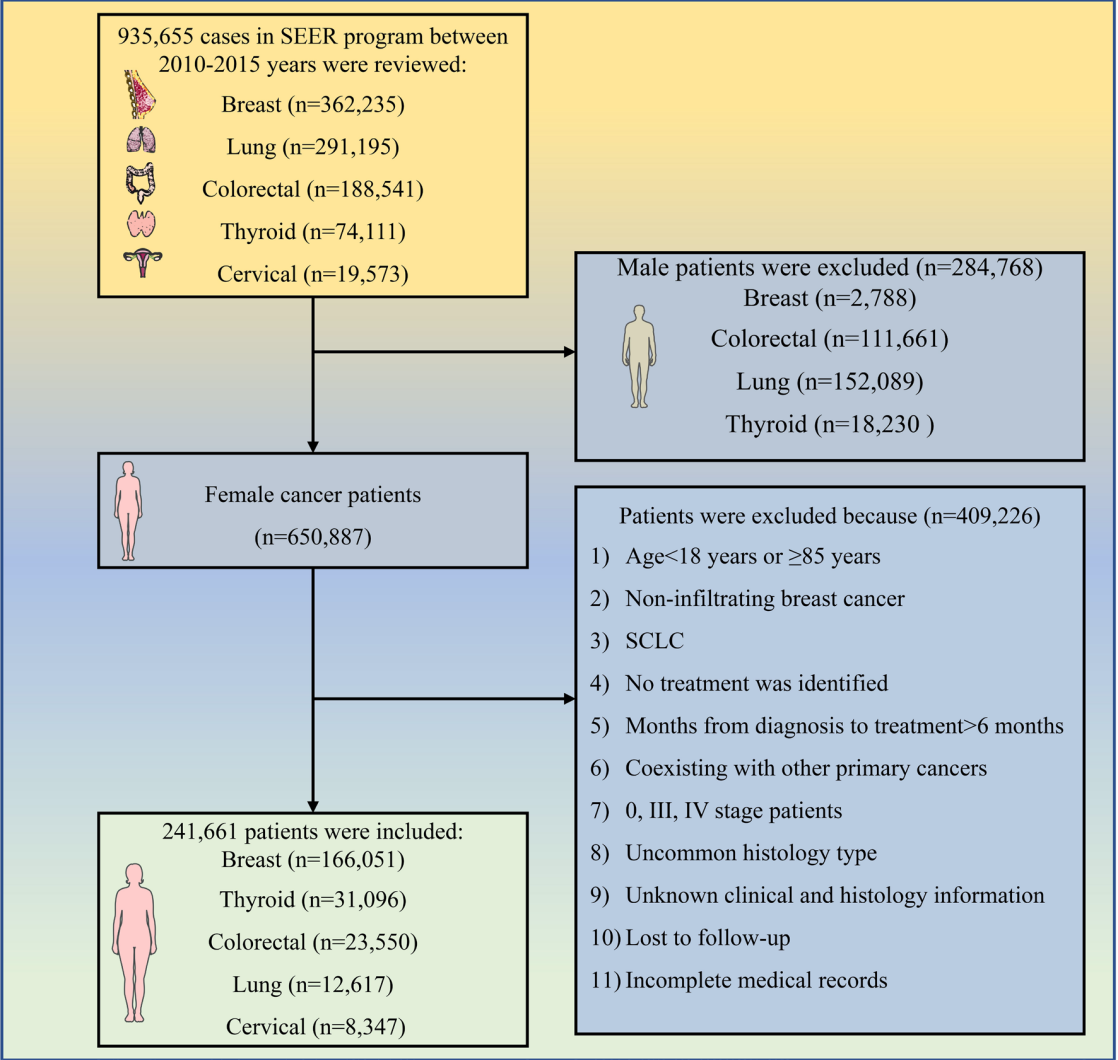
The patients’ selection process. *SEER* Surveillance, Epidemiology, and End Results, *SCLC* small cell lung cancer

We included females with early-stage cancer because these patients could derive more survival benefits from the different treatment modalities. Tumor staging was based on the guidelines of the American Joint Committee on Cancer Manual (Seventh edition) for tumors diagnosed from 2010 to 2015 and the International Federation of Gynecology and Obstetrics (FIGO) staging classification for cervical cancer. Female patients diagnosed between 2010 and 2015 with the following cancers were included in the analyses: stages I–II breast cancer, stages I–II non-small cell lung cancer (NSCLC), stages I–II colorectal cancer, stages I–II cervical cancer, and stages I–II differentiated thyroid cancer. These cancers were selected because they were the top five cancers with the highest incidence rates among women worldwide when the study was conducted. Patients who were eligible for definitive therapy using a variety of modalities were selected [1, 14]. Patients with the following characteristics were excluded from the study: age < 18 and ≥ 85 years old, diagnosed with other primary cancers, diagnosed with a T0 or Tis due to an unknown primary tumor site, a recipient of adequate treatment via tumor removal at the initial meeting [24], unknown race, unknown treatment history after the diagnosis was performed, or abnormal histology or presentation, such as inflammatory breast cancer, lost to follow-up, or incomplete medical records. Patients with treatment delays longer than 6 months were also excluded from the analysis because of the study’s small sample size and validity concerns. The flowchart of the patient selection process is presented in Fig. 1.

### Variables of interest

#### Demographic information

Basic information about the five cancers was reviewed before they were selected for the analyses. Patient’s demographic information, included age (calculated as a continuous variable), race (Hispanic, Non-Hispanic American Indian or Alaska Native, Non-Hispanic Asian or Pacific Islander, or Non-Hispanic Black or Non-Hispanic White), and year of diagnosis.

#### Clinicopathological information

The histological characteristics of the cancers were classified in accordance with the International Classification of Diseases for Oncology (Third Edition) (ICD-O-3) (Additional file 1: Table S1) and the Adolescents and Young Adults (AYA) site recode [25]. Five variables were collected and defined: (1) histological classification: NSCLC: adenocarcinoma and other subtypes; infiltrating breast cancer: infiltrating ductal carcinoma, infiltrating lobular carcinoma, and infiltrating duct mixed with lobular carcinoma; differentiated thyroid cancer: papillary thyroid carcinoma, follicular thyroid carcinoma, and follicular variant of papillary carcinoma; colorectal cancer: colon adenocarcinoma and rectum adenocarcinoma; cervical cancer: squamous carcinoma, adenocarcinoma, and adenosquamous carcinoma. (2) The differentiation grade: Grade I: well differentiated, Grade II: moderate differentiated, and Grade III/IV: poorly differentiated and undifferentiated, and unknown grade. (3) Tumor location: NSCLC: (i) upper lobe, (ii) middle lobe, (iii) lower lobe, and (iv) lung NOS; Infiltrating breast cancer: (i) nipple and center of the breast, (ii) upper and lower inner quadrant of the breast, (iii) upper and lower outer quadrant of the breast (iv) axillary tail of the breast, overlapping lesions of the breast and breast NOS; Colorectal carcinoma: (i) right-sided colon cancer including ascending colon, cecum, hepatic flexure, and the transverse colon, (ii) left-sided colon cancer including splenic flexure, descending colon, and sigmoid colon, (iii) the rectosigmoid junction and rectum, and (iv) large intestine, NOS. (4) TNM stage: T stage, N stage (N1a and N1b remained separated in differentiated thyroid cancer). (5) The cancer-associated treatment: surgery information, radiation therapy information, chemotherapy information.

#### Definitions of delays to treatment

No guidelines or consensus panels were available to define the cutoffs for delayed treatment following a diagnosis. Thus, an interval from diagnosis to the beginning of the initial treatment ≥ 1 month was considered a treatment delay, and an interval ≥ 3 months was considered a severe treatment delay, as defined in prior studies [21, 26]. Based on their monthly records, patients were stratified into three groups showing the lengths of their intervals from diagnosis to treatment: immediate treatment (< 1 month after diagnosis), intermediate delay (1–2 months delay), and prolonged delay (≥ 3 months) groups.

#### Supporting information

Supporting information is listed below: marital status at diagnosis was classified as (i) married, (ii) single, (iii) other, including divorced, separated, unmarried, domestic partner, or widowed, and (iv) unknown; the median household income was classified as (i) < $35,000, (ii) ≥ $35,000 and < $55,000, (iii) ≥ $55,000 and < $75,000, and (iv) ≥ $75,000); a facility or county was classified as (i) a county in a metropolitan area, (ii) a suburban or rural county adjacent to a metropolitan area, (iii) a suburban or rural county not adjacent to a metropolitan area, and (iv) an unknown area.

### Study outcomes

The primary purpose of this study was to evaluate the impact of prolonged delay intervals for treatment on the OS of female patients with cancer and an early tumor stage, given that the recurrence data on cancer was not available in the SEER program. The CSS and other variables accounting for basic information, socio-economic, and cancer-associated variables were further analyzed and served as secondary outcomes of this study.

### Statistical analysis

The Chi-square test was used to compare differences in categorical data among the three treatment interval groups of patients. Univariate and multivariate Cox regression analyses were performed to identify the independent prognostic factors in the OS of female patients with cancer. Based on the results of the multivariate Cox analysis, we extracted the hazard ratio coefficients of each variable to compute the predicted mean mortality outcomes of the female patients with early-stage cancers in the three subgroups, from the time of diagnosis to treatment. We analyzed the linear combination of the model’s estimate for each subgroup from the time of diagnosis to treatment, the mean age, and the highest proportion of other categorical confounders. The survival curves were estimated to determine the 10-year predicted mortality probabilities of each subgroup. A two-tailed p-value < 0.05 was considered significant. The results of the Kaplan–Meier analyses were plotted using the “survival,” “rms,” “survminer,” “ggplot,” and “foreign” packages of R software (R Foundation, Vienna, Austria, version 4.0.3, http://www.r-project.org).

## Results

### Demographic characteristics of the study population with different cancers

The study population from the SEER program between 2010 and 2015 consisted of 241,661 female patients with early-stage cancers, including 12,617 cases of NCSLC (5.2%), 166,051 cases of infiltrating breast cancer (68.7%), 31,096 cases of DTC (12.9%), 23,550 cases of colorectal cancer (9.7%), and 8347 cases of cervical cancer (3.5%). The mean ages of the study populations of five cancer cohorts were 68.11 years for the patients with NSCLC, 59.03 years for the patients with breast cancer, 45.3 years for the patients with DTC, 64.88 years for the patients with colorectal cancer, and 46.45 years for the patients with cervical cancer. The patients who identified their race as white accounted for a predominant proportion of the patients in the five cancer groups (ranging from 77.9 to 83.6%). A longer median follow-up (i.e., 1 month) was observed among patients with DTC (78 months, ranging from 0 to 119 months), whereas the shortest median follow-up time was observed in patients with NSCLC (57 months, ranging from 0 to 119 months). Most of the patients received surgical treatment, and less than half of them elected chemotherapy. A majority of patients started treatment within 1 month of their diagnosis for colorectal and DTC, but not for NSCLC, breast cancer, or cervical cancer. A detailed report of the clinical characteristics of patients across cancers is summarized in Table 1, and their cancer-specific characteristics are summarized in Additional file 1: Table S2.

**Table 1:** The demographic characteristics of early-stage female cancer patients with different time delay intervals

Variables	Subgroup	No. (%) of patients or median (range)				
		Lung (n = 12,617)	Breast (n = 166,051)	Thyroid (n = 31,096)	Colorectal (n = 23,550)	Cervical (n = 8347)
Follow-up time (m)	–	57 (0–119)	75 (0–119)	78 (0–119)	71 (0–119)	71 (0–119)
Time intervals (m)	–	1 (0–6)	1 (0–6)	0 (0–6)	0 (0–6)	1 (0–6)
Age, mean (SD), y	–	68.11 ± 9.67	59.03 ± 12.17	45.30 ± 13.87	64.88 ± 12.41	46.45 ± 13.11
Race	White	10,543 (83.6)	132,670 (79.9)	25,190 (81.0)	18,477 (78.5)	6504 (77.9)
	Black	1158 (9.2)	16,277 (9.8)	2285 (7.4)	2732 (11.6)	906 (10.9)
	Other^a^	916 (7.2)	17,105 (10.3)	3621 (11.6)	2341 (9.9)	936 (11.2)
	Other^b^	344 (2.7)	54,565 (32.9)	31,096 (100.0)	284 (1.2)	8346 (100.0)
Grade	I	2249 (17.8)	39,305 (23.7)	6939 (22.3)	2517 (10.7)	1410 (16.9)
	II	4936 (39.1)	72,688 (43.8)	1018 (3.3)	16,964 (72.0)	2929 (35.1)
	III/IV	3603 (28.5)	49,759 (29.9)	117 (0.4)	2813 (11.9)	2005 (24.0)
	Unknown	1829 (14.6)	4299 (2.6)	23,022 (74.0)	1256 (5.3)	2002 (24.0)
Stage^c^	I	9321 (73.9)	99,026 (59.6)	28,776 (92.5)	11,229 (47.7)	6566 (78.7)^e^
	II	3296 (26.1)	67,025 (40.4)	2320 (7.5)	12,321 (52.3)	1780 (21.3)^e^
T stage	T1	6569 (52.1)	112,311 (67.6)	22,350 (71.9)	6448 (27.4)	6566 (78.7)
	T2	4645 (36.8)	50,609 (30.5)	5593 (18.0)	4781 (20.3)	1780 (21.3)
	T3	1403 (11.1)	3131 (1.9)	2911 (9.3)	10,304 (43.8)	–
	T4	–	–	242 (0.8)	2017 (8.6)	–
N stage	N0	11,300 (89.6)	127,287 (76.7)	26,818 (86.2)	23,550 (100.0)	8346 (100.0)
	N1	1317 (10.4)	38,764 (23.3)	4278 (13.8)	–	0 (0.0)
Surgery	Performed	9731 (77.1)	163,955 (98.7)	31,096 (100.0)	23,100 (98.1)	6592 (79.0)
	Not performed	2886 (22.9)	2096 (1.3)	0 (0.0)	450 (1.9)	1754 (21.0)
Radiotherapy	Performed	3171 (25.1)	98,254 (59.2)	12,347 (39.7)	2703 (11.5)	3529 (42.3)
	No/unknown	9446 (74.9)	67,797 (40.8)	18,749 (60.3)	20,847 (88.5)	4817 (557.7)
Chemotherapy	Performed	2564 (20.3)	65,930 (39.7)	–	4531 (19.2)	2799 (33.5)
	No/unknown	10,053 (79.7)	100,121 (60.3)	–	19,019 (80.8)	5547 (66.5)
Diagnosis year	2010	1955 (15.5)	24,126 (14.5)	4669 (15.0)	3981 (16.9)	1434 (17.2)
	2011	2033 (16.1)	25,978 (15.7)	4921 (15.8)	3891 (16.5)	1353 (16.2)
	2012	2046 (16.2)	27,234 (16.4)	5142 (16.5)	4020 (17.1)	1399 (16.8)
	2013	2168 (17.2)	28,304 (17.0)	5279 (17.0)	3827 (16.2)	1307 (15.7)
	2014	2136 (16.9)	29,409 (17.7)	5481 (17.7)	4012 (17.0)	1401 (16.9)
	2015	2280 (18.1)	31,000 (18.7)	5604 (18.0)	3819 (16.2)	1452 (17.4)
Marital status	Married	5505 (43.6)	98,063 (59.0)	18,735 (60.2)	11,321 (48.1)	3824 (45.8)
	Single	1546 (12.3)	23,370 (14.1)	6958 (22.4)	3452 (14.7)	2441 (29.2)
	Other^c^	4953 (39.3)	36,892 (22.2)	3713 (12.0)	7267 (30.9)	1610 (19.3)
	Unknown	613 (4.8)	7726 (4.7)	1690 (5.4)	1510 (6.4)	471 (5.6)
Income^d^, $	< 35k	342 (2.7)	2481 (1.5)	483 (1.6)	548 (2.3)	173 (2.1)
	35k to < 55k	3358 (26.6)	34,663 (20.9)	6275 (20.2)	5868 (24.9)	2057 (24.6)
	55k to < 75k	5621 (44.6)	78,176 (47.1)	15,033 (48.2)	10,973 (46.6)	4000 (47.9)
	> 75k	3296 (26.1)	50,731 (30.5)	9305 (30.0)	6161 (26.2)	2116 (25.3)
County	Metropolitan	10,887 (86.3)	149,035 (89.8)	28,127 (90.5)	20,315 (86.3)	7403 (88.7)
	Near metropolitan	960 (7.6)	9785 (5.9)	1628 (5.2)	1764 (7.5)	518 (6.2)
	Not near metropolitan	748 (5.9)	6997 (4.2)	1302 (4.2)	1399 (5.9)	412 (4.9)
	Unknown	22 (0.2)	234 (0.1)	39 (0.1)	72 (0.3)	13 (0.2)

^a^Other: defined as the Asian/Pacific Islander and American Indian/Alaska Native

^b^Stage: 7th edition of TNM stage classification by the American Joint Committee on Cancer

^c^Other: Divorced, separated, windowed, unmarried or domestic partner

^d^Income: median household income inflation adj to 2019

^e^FIGO stage

### Trends in the times from diagnosis to treatment of patients with different cancers

The intervals showing the times from diagnosis to treatment of patients with different cancers are presented in Table 2. More than half of the patients in the study with DTC (70.8% in stage I and 69.2% in stage II) or NSCLC (54.7% in stage I and 62.4% in stage II) experienced intermediate delays in treatment initiation after they were diagnosed with cancer. Moreover, the treatment delay was universal among the patients diagnosed with NSCLC, compared to the patients with the other four types of cancers. Overall, only a small proportion of early-stage patients experienced long delays in receiving treatment for cancer (ranging from 1.6 to 12.0%), except for the patients with NSCLC.

**Table 2:** Trends in time from diagnosis to treatment in female cancer patients with different stages

Variables	No. (%) of patients			P*
	< 1 month (immediate treatment)	1–2 months (intermediate delay)	3–6 months (long delay)	
NSCLC				
Stage I	2896 (31.1)	5104 (54.7)	1321 (14.2)	**< 0.001**
Stage II	764 (23.2)	2056 (62.4)	476 (14.4)	
IBC				
Stage I	23,424 (23.7)	70,132 (70.8)	5470 (5.5)	**< 0.001**
Stage II	16,576 (24.7)	46,367 (69.2)	4082 (6.1)	
DTC				
Stage I	19,172 (66.6)	7994 (27.8)	1610 (5.6)	**0.043**
Stage II	1589 (68.5)	590 (25.4)	141 (6.1)	
Colorectal cancer				
Stage I	6791 (60.5)	4099 (36.5)	339 (3.0)	**< 0.001**
Stage II	6491 (52.7)	5474 (44.4)	356 (2.9)	
Cervical cancer				
Stage I	3369 (51.3)	2652 (40.4)	545 (8.3)	**< 0.001**
Stage II	425 (23.9)	1141 (64.1)	214 (12.0)	

The tumor stage refers to the TNM stage classification in the 7th edition

*NSCLC* non-small cell lung cancer, *IBC* infiltrating breast cancer, *DTC* differentiated thyroid carcinoma

Bold values indicate statistical significance (*p* < 0.05)

*Pearson’s Chi-squared test

### Impact of treatment delays on OS: results of the multivariate Cox analysis

After adjusting for other confounders, significantly worse survival patterns were observed in patients with stage I NSCLC [_adjusted_Hazard Ratio (HR) = 1.11, 95% Confidence Interval (CI): 1.01–1.23, p = 0.044]. Long delays in the initiation of treatment were associated with worse survival, compared with no delays in the immediate initiation of treatment (_adjusted_HR = 1.23, 95% CI 1.11–1.37, p < 0.001). In contrast, better survival outcomes were observed in patients with intermediate delays, compared with patients who received immediate treatment, regardless of their stage at presentation (HR = 0.92, 95% CI 0.87–0.97, p = 0.003 in stage I patients and _adjusted_HR = 0.93, 95% CI 0.88–0.97, p = 0.002 in stage II patients). Moreover, a positive association was found with the OS of patients with early-stage colorectal cancer and intermediate delays in treatment (_adjusted_HR = 0.83, 95% CI 0.75–0.92, p < 0.001 in stage I patients and _adjusted_HR = 0.70, 95% CI 0.65–0.75, p < 0.001 in stage II patients). However, no significant association was found between the treatment delays and OS in patients with early-stage DTC or cervical cancer (Table 3). Similar results were observed when the treatment delay intervals were treated as continuous variables (_adjusted_HR = 1.04, 95% CI 1.01–1.06, p = 0.010 in stage I NSCLC and _adjusted_HR = 1.03, 95% CI 1.00–1.06, p = 0.029 in stage I breast cancer) (Additional file 1: Table S3-1).

**Table 3:** Univariate and Multivariable analyses of the association of treatment delay intervals with overall survival

Cancer	Subgroup^d^	Model 1		Model 2^a^	
		HR^b^ (95% CI)	*P*	HR (95% CI)	*P*
NSCLC					
Stage I	Intermediate	1.47 (1.36–1.58)	**< 0.001**	1.04 (0.95–1.13)	0.349
	Long	2.05 (1.85–2.25)	**< 0.001**	1.11 (1.01–1.23)	**0.044**
Stage II	Intermediate	1.20 (1.07–1.33)	**0.001**	0.97 (0.87–1.09)	0.699
	Long	1.48 (1.28–1.70)	**< 0.001**	1.00 (0.86–1.16)	0.995
IBC					
Stage I	Intermediate	0.91 (0.86–0.96)	**0.001**	0.92 (0.87–0.97)	**0.003**
	Long	1.20 (1.08–1.34)	**< 0.001**	1.23 (1.11–1.37)	**< 0.001**
Stage II	Intermediate	0.86 (0.82–0.90)	**< 0.001**	0.93 (0.88–0.97)	**0.002**
	Long	1.10 (1.01–1.20)	**0.038**	1.09 (0.99–1.19)	0.059
DTC					
Stage I	Intermediate	0.43 (0.33–0.57)	**< 0.001**	0.70 (0.53–0.93)	**0.012**
	Long	0.76 (0.49–1.19)	0.236	1.09 (0.70–1.70)	0.711
Stage II	Intermediate	0.74 (0.43–1.29)	0.295	0.73 (0.41–1.31)	0.300
	Long	0.21 (0.03–1.50)	0.120	0.22 (0.03–1.61)	0.138
Colorectal cancer					
Stage I	Intermediate	0.97 (0.88–1.06)	0.503	0.83 (0.75–0.92)	**< 0.001**
	Long	1.60 (1.29–2.00)	**< 0.001**	1.21 (0.97–1.52)	0.090
Stage II	Intermediate	0.71 (0.66–0.76)	**< 0.001**	0.70 (0.65–0.75)	**< 0.001**
	Long	1.32 (1.11–1.57)	**0.001**	0.87 (0.72–1.04)	0.120
Cervical cancer					
Stage I	Intermediate	1.75 (1.48–2.08)	**< 0.001**	1.08 (0.90–1.30)	0.421
	Long	1.70 (1.28–2.26)	**< 0.001**	0.84 (0.63–1.14)	0.282
Stage II	Intermediate	1.04 (0.85–1.27)	0.713	0.92 (0.74–1.14)	0.436
	Long	1.15 (0.86–1.54)	0.340	1.01 (0.75–1.37)	0.949

*HR* hazard ratio, *CI* confidence interval, *NSCLC* non-small cell lung cancer, *IBC* infiltrating breast cancer, *DTC* differentiated thyroid carcinoma

^a^Model 2 was adjusted by the age, race, tumor location (exception of DTC, cervical cancer), differentiated grade; histology (exception of colorectal cancer), T stage, N stage (exception of NSCLC in stage I, DTC in stage II, colorectal cancer), surgery (exception of DTC), radiotherapy, chemotherapy (exception of DTC), marital status, income, and molecular subtype (only for breast cancer)

^b^HR: compared with immediate treatment initiation

^c^Cervical cancer: FIGO stage

^d^Subgroup: immediate: < 1 month, intermediate delayed: ≥ 1 and ≤ 2 months, and long-delayed: ≥ 3 months

Bold values indicate statistical significance (*p* < 0.05)

### Impact of treatment delays on CSS: results of the multivariate Cox analysis

Similarly, a protective role of intermediate delays was observed in the treatment of stage I breast cancer (_adjusted_HR = 0.88, 95% CI 0.79–0.98, p = 0.018) and stage II breast cancer (_adjusted_HR = 0.91, 95% CI 0.85–0.96, p = 0.002). The beneficial role of delays in treatment was only observed in patients with stage II colorectal cancer with intermediate delays (_adjusted_HR = 0.64, 95% CI 0.57–0.71, p < 0.001) and those with prolonged delays (_adjusted_HR = 0.67, 95% CI 0.51–0.86, p = 0.002). However, intermediate delays significantly impaired the CSS of patients with stage I cervical cancer (_adjusted_HR = 1.31, 95% CI 1.02–1.68, p = 0.032). No association was found between the treatment delays and CSS of patients with NSCLC or DTC (p > 0.05) (Table 4). When the treatment delay intervals were calculated as continuous variables, the protective role of delayed treatment initiation was found only in patients with stage II colorectal cancer (_adjusted_HR = 0.83, 95% CI 0.78–0.88, p < 0.001) (Additional file 1: Table S3-2).

**Table 4:** Univariate and Multivariable analyses of the association of treatment delay intervals with cancer-specific survival (CSS)

Cancer	Subgroup^d^	Model 1		Model 2^a^	
		HR^b^ (95% CI)	*P*	HR (95% CI)	*P*
NSCLC					
Stage I	Intermediate	1.47 (1.32–1.63)	**< 0.001**	0.98 (0.88–1.10)	0.793
	Long	2.03 (1.78–2.33)	**< 0.001**	1.04 (0.90–1.20)	0.575
Stage II	Intermediate	1.17 (1.04–1.32)	**0.011**	0.96 (0.85–1.09)	0.525
	Long	1.32 (1.12–1.56)	**0.001**	0.91 (0.77–1.08)	0.292
IBC					
Stage I	Intermediate	0.86 (0.77–0.95)	**0.004**	0.88 (0.79–0.98)	**0.018**
	Long	0.97 (0.82–1.21)	0.798	0.96 (0.77–1.20)	0.729
Stage II	Intermediate	0.81 (0.76–0.86)	**< 0.001**	0.91 (0.85–0.96)	**0.002**
	Long	0.96 (0.86–1.08)	0.546	1.02 (0.91–1.15)	0.703
DTC					
Stage I	Intermediate	1.21 (0.36–4.00)	0.759	1.10 (0.28–4.33)	0.890
	Long	1.56 (0.19–12.54)	0.676	1.28 (0.14–11.22)	0.820
Stage II	Intermediate	0.03 (0.00–174.28)	0.420	0.03 (0.00–19.91)	0.293
	Long	0.03 (0.00–2.17 × 10^6^)	0.698	0.10 (0.00–1.19 × 10^7^)	0.664
Colorectal cancer					
Stage I	Intermediate	1.30 (1.07–1.56)	**0.006**	0.87 (0.71–1.07)	0.192
	Long	2.50 (1.70–3.67)	**< 0.001**	1.13 (0.75–1.71)	0.555
Stage II	Intermediate	0.71 (0.64–0.78)	**< 0.001**	0.64 (0.57–0.71)	**< 0.001**
	Long	1.26 (0.99–1.61)	0.059	0.67 (0.51–0.86)	**0.002**
Cervical cancer					
Stage I	Intermediate	2.20 (1.75–2.77)	**< 0.001**	1.31 (1.02–1.68)	**0.032**
	Long	2.30 (1.61–3.30)	**< 0.001**	1.25 (0.85–1.82)	0.255
Stage II	Intermediate	1.10 (0.86–1.40)	0.451	1.01 (0.78–1.32)	0.918
	Long	1.18 (0.83–1.68)	0.345	1.09 (0.75–1.57)	0.649

*HR* hazard ratio, *CI* confidence interval, *NSCLC* non-small cell lung cancer, *IBC* infiltrating breast cancer, *DTC* differentiated thyroid carcinoma

^a^Model 2 was adjusted by the age, race, tumor location (exception of DTC, cervical cancer), differentiated grade; histology (exception of colorectal cancer), T stage, N stage (exception of NSCLC in stage I, DTC in stage II, colorectal cancer), surgery (exception of DTC), radiotherapy, chemotherapy (exception of DTC), marital status, income, and molecular subtype (only for breast cancer)

^b^HR: compared with immediate treatment initiation

^c^Cervical cancer: FIGO stage

^d^Subgroup: immediate: < 1 month, intermediate delayed: ≥ 1 and ≤ 2 months, and long-delayed: ≥ 3 months

Bold values indicate statistical significance (*p* < 0.05)

### Prediction of the 10-year OS of patients with different treatment delay intervals

**Fig. 2:**
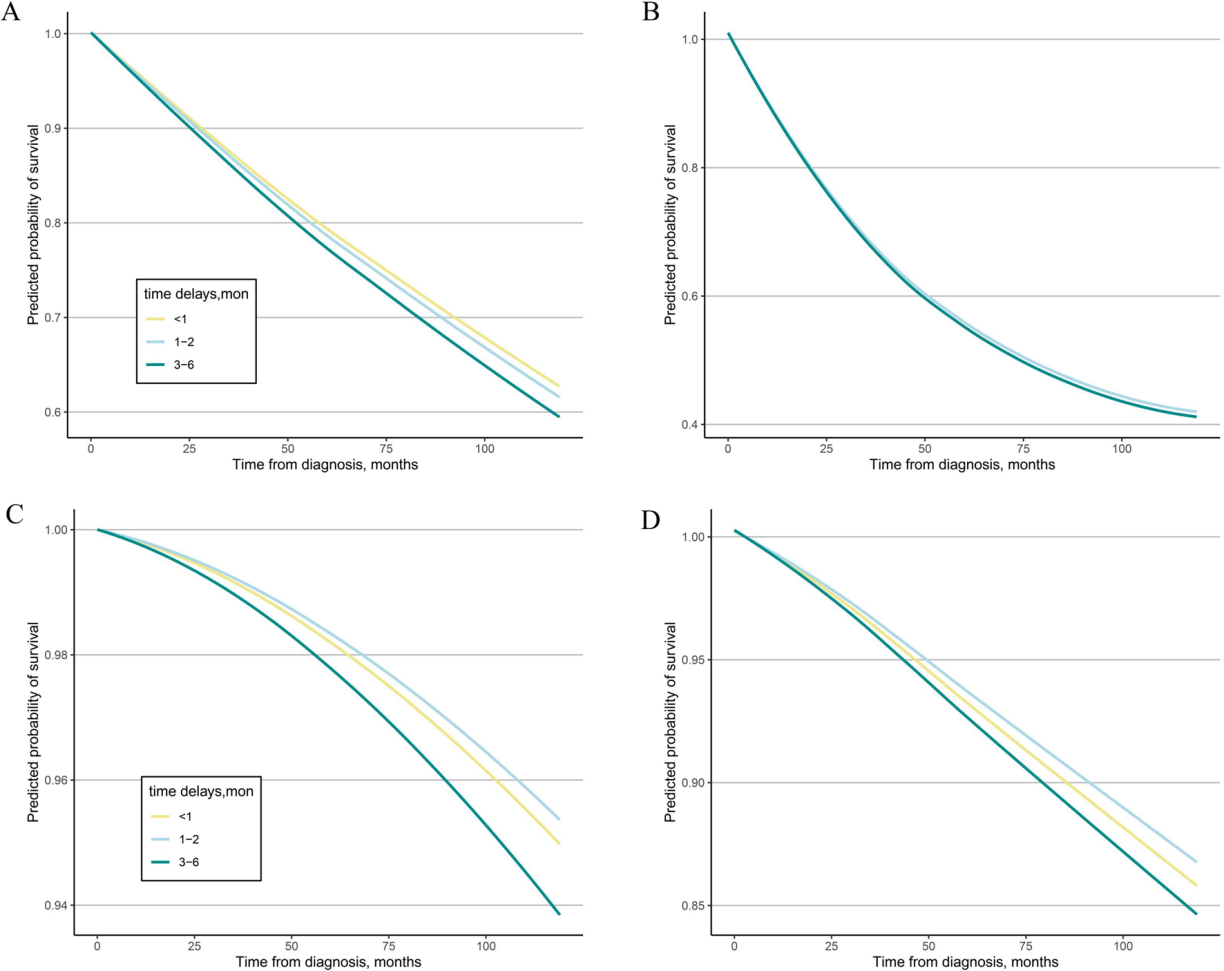
The predicted 10-year survival in female cancer patients with different time intervals to treatment. **A** stage I NSCLC, **B** stage II NSCLC, **C** stage I infiltrating breast cancer, **D** stage II infiltrating breast cancer, *NSCLC* non-small cell lung cancer

**Fig. 3:**
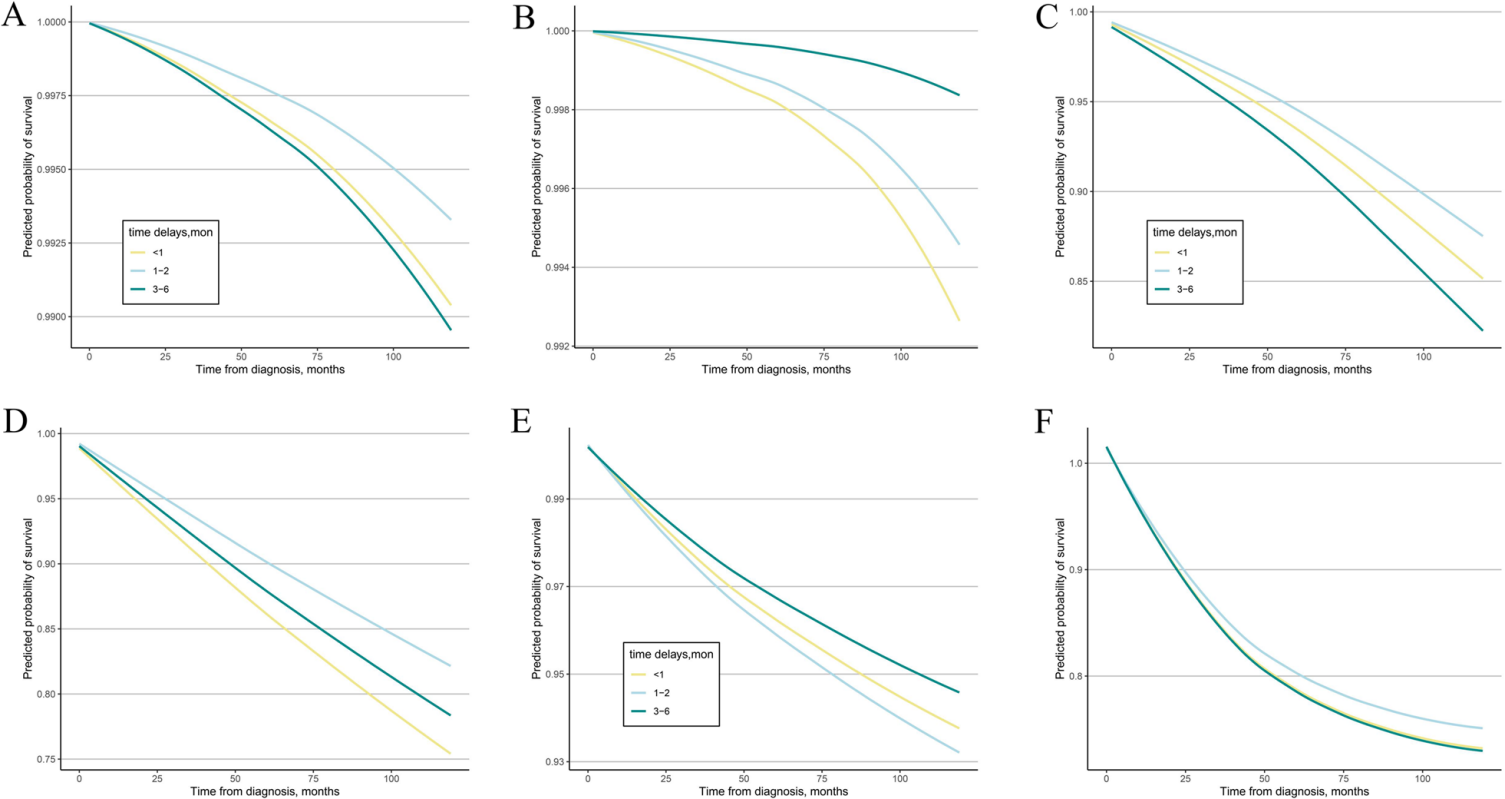
The predicted 10-year survival in female cancer patients with different time intervals to treatment. **A** stage I DTC, **B** stage II DTC, **C** stage I colorectal cancer, **D** stage II colorectal cancer, **E** stage I cervical cancer, **F** stage II cervical cancer. *DTC* differentiated thyroid carcinoma

Based on the results of the multivariate Cox analysis, we further analyzed the 10-year predicted OS patterns of the three groups of patients with different treatment delay intervals (i.e., the immediate treatment, intermediate delay, and prolonged delay groups). The 10-year OS probabilities were 63.5%, 62.4%, and 60.3% in the immediate treatment, intermediate delay, and long delay groups with stage I NSCLC, respectively (Fig. 2A). The 10-year OS probabilities were 41.1%, 41.9%, and 41.1% in the immediate treatment, intermediate delay, and prolonged delay groups with stage II NSCLC, respectively (Fig. 2B). The predicted 10-year OS was 95.1%, 95.5%, and 94.0% in the immediate treatment, intermediate delay, and prolonged delay groups with stage I breast cancer, respectively, (Fig. 2C) and 86.0%, 86.9%, and 84.8% in the immediate treatment, intermediate delay, and long delay groups with stage II breast cancer, respectively (Fig. 2D). A favorable OS rate was found in the three subgroups with DTC (the predicted 10-year OS was 99.0%, 99.3%, and 98.9% in the immediate treatment, intermediate delay, and long delay groups with stage I cancer, respectively, and 99.0%, 99.3%, and 99.8% in the immediate treatment, intermediate delay, and long delay groups with stage II cancer, respectively) (Fig. 3A, B). The predicted 10-year OS of patients with stage I colorectal cancer was 85.6%, 87.9%, and 82.8% in the immediate treatment, intermediate delay, and long delay groups, respectively, (Fig. 3C) and 75.6%, 82.3%, and 78.5% in the immediate treatment, intermediate delay, and long delay groups with stage II cancer (Fig. 3D), respectively. The 10-year OS probabilities (93.7%, 93.2%, and 94.5%) were similar among the immediate treatment, intermediate delay, and long delay groups in stage I patients, respectively, and the OS probabilities (71.2%, 73.2%, and 71.0%) were similar among the immediate treatment, intermediate delay, and long delay groups in stage II patients, respectively (Fig. 3E, F).

## Discussion

Cancer-associated treatment delays were not uncommon during the COVID-19 pandemic, especially in patients with high incidence cancers [15, 27–29]. Treatment delays increased anxiety and depression in patients with cancer, especially female patients, due to concerns about becoming infected with COVID-19 or because of strict nationwide policies [15]. Our model predicted an increasing number of deaths among patients with cancers, such as breast cancer, colorectal cancer, lung cancer, and esophageal cancer in the beginning and after the end of the COVID-19 pandemic [30]. Therefore, we thought an investigation of the impact of treatment delays on the long-term survival of patients with the most common female cancers could help physicians develop interventions for this population that were more rational. In the present study, we examined discrepancies in the impact of delays in the initiation of treatment on the survival of females with five different cancers.

In this large population-based study, the probability of OS was found to be worse (_adjusted_HR = 1.23, p < 0.001) among patients with stage I breast cancer. Unlike patients whose treatment initiation was timely, a borderline negative association was found between long-term treatment delays and OS among patients with stage II cancer (_adjusted_HR = 1.09, 95% CI 0.99–1.19, p = 0.059). However, treatment delays did not impair the CSS of this population, regardless of the cancer stage at diagnosis. An observational study conducted using the National Cancer Database (NCDB) determined that the outcomes of even stage I patients could be influenced by a delay in the initiation of treatment. A delay in treatment from 61 to 120 days after diagnosis was associated with a 1.3% and 2.3% increased mortality after 5 years and 10 years, respectively [24]. In contrast, the results of a study conducted in Singapore by Ho et al. did not find any significant differences in long-term survival between patients with non-invasive breast cancer who received treatment after a 90-day wait-time interval and those who received treatment within the 90-day interval. Notably, the ethnic composition of the participants was different in the two studies (White and Asian). Recent studies have confirmed inconsistent survival patterns among patients with cancer of different ethnicities and races [14, 31]. Specifically, the survival probability of Black patients has been reported to be relatively lower than the survival of Asian/Pacific Islanders. Although underlying mechanisms of survival are under investigation, factors such as lifestyle and socioeconomic status are thought to play a pivotal role in survival [31, 32]. Nevertheless, the results of our analysis support the importance of timely definitive treatment, even for early-stage breast cancer. Patients with ductal carcinoma in situ breast cancer (DCIS) were not included in this study because of their limited number of cases and most of these patients had a satisfactory prognosis after surgical intervention. Minami et al. found that prolonged wait times for operations to treat DCIS were associated with a slight increase in pathological upstaging in the patients, although this delay did not affect their OS [33].

To date, the conclusions of investigations of the association between prolonged wait times and the survival of patients with NSCLC are still debatable [6, 34–36]. As one review concluded, the impact of treatment delays was lowest for subcentimeter nodules, and probably the highest in stage II disease. However, in an evidence-based large-scale study from Taiwan (42,962 cases), the authors reported a negative impact of treatment delays on patients with whole-stage NSCLC; those with late-stage tumors or longer wait-time intervals showed even larger increases in mortality [37]. Compared with the Taiwan cohort, the females in our study with NSCLC showed a paradoxical result. An increased risk of mortality due to treatment delays was observed in the stage I patients with NSCLC (_adjusted_HR = 1.11, 95% CI 1.01–1.23, p = 0.044), compared with the patients who were treated immediately. These results were found for the OS group but not for CSS. Cone et al. found an increased risk of all-cause mortality among patients with stage I NSCLC from a large cancer database in the US [24], regardless of sex. However, this phenomenon was not present in stage II patients because the survival curves did not show a significant discrete distribution among the different treatment delays of the subgroups, as described. The explanations for this difference could reflect a potential bias. First, the definitions of treatment delays varied among existing studies, which could have led to inconsistencies in reported trends. The study populations and sample sizes among the various studies might have also contributed to this difference. Although existing studies have limitations, their results mostly support the association of timely treatment and better OS in patients with non-metastatic stage NSCLC [34]. Whether this finding applies to CSS requires further evaluation. Recommendations derived from more robust evidence could improve future research, policies, and healthcare practices.

Although different strategies to treat stages I-II DTC can be implemented, surgical intervention is the pivotal first-line treatment modality. Thus, we only included patients who underwent surgical resection in the present study. As expected, we observed no significant effects of treatment delays on the long-term survival of patients with early-stage cancer. In contrast, a significantly higher probability of survival was observed in stage I patients with intermediate delays in treatment (_adjusted_HR = 0.70, 95% CI 0.53–0.93, p = 0.012). In recent years, DTC has been regarded as a type of indolent cancer and the consensus of experts in developed countries suggests that active surveillance could be a safe and effective strategy for dealing with DTC in low-risk patients [38]. Up to now, few studies have evaluated the influence of treatment delays on DTC survival. However, a study on this topic by Fligor et al. determined that delayed surgery among patients with papillary thyroid carcinoma was associated with reduced OS [39]. Some differences among these studies should be acknowledged. First, the median follow-up time in the study by Fligor et al. was 55 months, which was relatively shorter than the follow-up of our study (78 months). Second, longer intervals were examined in their study (90 days), whereas 90 days was further divided into two subgroups in our study. In addition, their subgroup analysis was based on the T stage, while our study was based on the tumor stage. Our results support the application of an acceptable wait time that is safe for the initiation of treatment for early-stage female patients with cancer. Future studies should be conducted to analyze the impact of treatment delays on the risk of disease progression and recurrence-free survival in this population, which could enhance the evidence supporting the use of active surveillance for these patients.

No significant difference among patients with stage I colorectal cancer was found between those with prolonged treatment delays and their counterparts who received immediate treatment. However, a trend persisted (_unadjusted_HR = 1.60, p < 0.001; _adjusted_HR = 1.21, p = 0.090): two studies on patients from the NCDB with different cutoffs for wait-time intervals (40 days and 60 days) revealed significantly worse outcomes among the patients with curable-stage colorectal cancer and a delay in the initiation of treatment [24, 40]. However, a recent high-volume single-center study in China did not find any negative effects of prolonged preoperative wait times on the prognosis of outcomes (including OS and disease-free survival) of patients with stages I-III colorectal cancer [41]. However, an earlier meta-analysis reported that inconsistent findings were frequent in studies on colorectal cancer, with the majority of findings showing “no association” [6]. Of note, when our study population was further divided into two subgroups (immediate treatment: < 1 month and intermediate delay: ≥ 1 and < 3 months), intermediate delays in treatment was associated with better OS (_adjusted_HR = 0.83, 95% CI 0.75–0.92, p < 0.001) in stage I patients and (_adjusted_HR = 0.70, 95% CI 0.65–0.75, p < 0.001) in stage II patients, and CSS rates (_adjusted_HR = 0.64, 95% CI 0.57–0.71, p < 0.001) in stage II patients. Nevertheless, we cannot interpret this association as presented, because whether an intermediate delay was caused by a pre-treatment evaluation or a referral to a specialized center providing better clinical care remains unknown. Thus, delaying treatment of the patients with colorectal cancer for relatively prolonged time intervals is questionable by deserves further exploration.

Recent systematic reviews and meta-analyses have been conducted on the association between delayed initiation of treatment and the survival time of patients with cervical cancer [42]. To the best of our knowledge, this is the largest nationwide cohort to examine the impact of treatment delay intervals on the long-term survival of patients with early-stage cervical cancer. Our results showed that delayed initiation of treatment did not adversely affect the OS of patients with early-stage cervical cancer, but it increased the risk of CSS in stage I patients with intermediate treatment delays. In a review of the recent literature on cervical cancer, the disparity in survival patterns was found to be an effect of prolonged wait times for different treatment modalities [21, 22, 43–45]. Most of the studies on this topic were limited to single-centers or had small samples. For instance, Umezu et al. [44] (n = 177 cases), Perri et al. [45] (n = 321 cases), and Matsuo et al. 41 (n = 217 cases) found that a delayed time from the initial visit (and diagnosis) to the intervention did not impair outcomes of patients with cervical cancer. However, evidence from a large population-based study in Asia [21] showed that patients with early-stage cervical cancer and longer wait-time intervals (especially those exceeding 180 days) exhibited a significantly lower probability of OS than the patients who received treatment within 90 days did. Hence, the different cutoffs of the wait-time intervals (which ranged from 14 to 90 days) might have contributed to this divergence in OS rates.

The conclusions drawn inform the evaluations of the impact of delayed adjuvant therapy initiation on the survival of patients with cervical cancer were consistent. Noh et al. [43] reported that wait-time intervals for definitive concurrent chemoradiation had a significant influence on the OS of patients with cervical cancer when the cutoff wait-time interval was 14 days (as a categorical variable: HR = 1.53, p = 0.018; and as a continuous variable: HR = 1.023/per day, p = 0.007). Similarly, the delayed initiation of adjuvant radiotherapy also impaired the OS of patients with early-stage cervical cancer [22]. Thus, delayed time to treatment may have had a greater impact on the postoperative adjuvant therapy prior to the initial treatment. Robust evidence derived from comprehensive analyses in the future (such as uniform cutoffs of intervals, longer follow-ups, and multicenter datasets) should be considered to help clinicians improve their therapeutic plans for patients with cervical cancer.

Although some of the cancers and stages mentioned in the current study showed better OS and CSS patterns in the intermediate treatment delay group, the results need to be interpreted carefully and evaluated further. A relatively prolonged time from diagnosis to treatment might not impair the prognosis of most patients with cancer after adjusting for numerous confounders, revealing that cancer-specific characteristics are more pivotal prognostic factors than wait-time intervals between diagnosis and treatment.

Accumulative evidence suggests that a multi-level social network is associated with improved survival among patients with cancer. To date, many scholars believe that the connection between marital status and cancer survival reflects social support. Spouses motivate patients to seek regular medical care, which can lead to an earlier diagnosis and treatment of a disease. Patients with cancer receive encouragement from their spouses to continue to follow up with their treatment and to seek support from other survivors of cancer [46–49], and previous studies have examined the association of marital status with breast cancer risk and survival [49–51]. In an earlier study, researchers showed that unmarried women were at increased risk for death from breast cancer, which might be attributed to their decreased social support and social networks [52]. According to a report by Parise et al., the survival patterns of married white women and female Asian/Pacific Islanders with triple-negative breast cancer improved after adjusting for confounders such as age, stage, tumor grade, and treatment [53].

In the present study, married patients also had shorter wait-time intervals from diagnosis to treatment, regardless of their cancer stage and subtype. Furthermore, a significantly higher probability of long-term OS was found in married patients with stage I NSCLC (Additional file 1: Table S4), breast cancer (Additional file 1: Table S5), differentiated thyroid cancer (Additional file 1: Table S6), and colorectal cancer (Additional file 1: Table S7), compared with single or divorced patients. However, this effect was not observed in patients with early-stage cervical cancer (Additional file 1: Table S8). The insurance data in our study was missing; therefore, the median household income was used as a reference for an indirect measure of insurance status. Similar to the findings of marital status, early-stage patients with high incomes (> $75,000/year) showed significantly better survival outcomes, compared with low-income patients (< $35,000/year), especially patients with NSCLC, breast cancer, and colorectal cancer. The underlying mechanisms in the associations between socioeconomic status and cancer outcomes, as well as its clinical influence, need further exploration. Nevertheless, our results showed that appropriate psychosocial care and support helped female patients cope with their cancer diagnosis and treatment, especially low-income, divorced, and unmarried subpopulations.

The present study has strengths that should be mentioned. First, it was conducted using a nationwide large-scale database. Our results partially confirmed the conclusions of previous studies in the field of breast cancer, NSCLC, and colorectal cancer, and our study provides new findings on cervical and thyroid cancers among female patients. Second, we focused on cancers with the highest incidence rates among females worldwide to help researchers understand the impact of wait-time intervals on the prognosis of patients with early-stage cancers, especially female-specific or predominate cancers.

To date, most of the evidence supporting the association between the time to treatment initiation and survival is based on data from cancer registries from developed countries. Thus, future research should be conducted in the middle- or low-income countries, where patients with cancer are more vulnerable to disease progression due to treatment delays. Careful scrutiny and comprehensive evaluations of the time from diagnosis to the initiation of treatment within institutions are likely to have far-reaching effects, which could help public health administrators make tailored decisions on treatment deferral in resource-limited regions due to pandemics and facilitate the process of global health missions.

The present study has several limitations that need to be addressed. First, this is a retrospective observational study with selection bias, unbalanced baseline characteristics, and other potential confounders. Second, information about the patient’s characteristics, including lifestyle, educational level, insurance status, Charlson-Deyo comorbidity index, mental health, and medical knowledge, which might have affected their prognosis [15, 28, 54], were not available in the SEER database. Third, we only included cancers with the highest incidence rates in females for the analysis. Thus, future studies should include more cancer subtypes to reach more comprehensive and robust conclusions for clinical decision-making. Furthermore, a detailed record of the reasons for the treatment delays was unavailable in the present database. This information is crucial for research on this important topic and for reducing the progression of cancers due to treatment delays.

## Conclusion

Our findings indicate that prolonged delays in treatment initiation (within 6 months) have a limited negative impact on the survival of patients with early-stage NCSLC, infiltrating breast cancer, DTC, colorectal cancer, and cervical cancer. Whether these results can be used as evidence supporting a more comprehensive pre-treatment evaluation needs further exploration and validation, and patients' preferences and anxiety levels should be considered.

## Supplementary Information

**Additional file 1: Table S1.** The code for International Classification of Disease for Oncology third edition of the present study. **Table S2.** The cancer-specific characteristics of five different cancers. **Table S3.** Univariate and Multivariable Cox analyses of the association of treatment delay intervals with overall survival and cancer-specific survival (treatment delay intervals were calculated as continuous variables). **Tables S4–S8.** Multivariable analyses of the association of treatment delay intervals with overall survival in five different cancers.

## Data Availability

The data that support the findings of this study are available on request from the corresponding author. These data were derived from the following resources available in the public domain: the Surveillance, Epidemiology, and End Results (SEER) Program at (https://seer.cancer.gov/data-software/).
