# Supplementary material for: Survival outcomes following treatment delays among patients with early-stage female cancers: a nationwide study
Source: J Transl Med. 2022 Dec 3;20:560. doi: 10.1186/s12967-022-03719-7 (PMC9719121; doi:10.1186/s12967-022-03719-7)
Supplement: Supplementary file 1 — Additional file 1: Table S1. The code for International Classification of Disease for Oncology third edition of the present study. Table S2. The cancer-specific characteristics of five different cancers. Table S3. Univariate and Multivariable Cox analyses of the association of treatment delay intervals with overall survival and cancer-specific survival (treatment delay intervals were calculated as continuous variables). Tables S4–S8. Multivariable analyses of the association of treatment delay intervals with overall survival in five different cancers. [file 12967_2022_3719_MOESM1_ESM.pdf]

Table S1. The code for International Classification of Disease for Oncology third edition in the present study.

Cancer types	ICD-O-3
Non-small cell lung cancer	8046/3, 8070/3-8074/3, 8084/3,8140/3, 8250/3 ,8252/3, 8253/3, 8255/3, 8480/3, 8560/3
Infiltrating breast cancer	8500/3, 8520/3-8524/3
Colorectum cancer	8140/3, 8143/3, 8145/3, 8201/3, 8210/3, 8211/3, 8213/3, 8220/3, 8221/3, 8255/3, 8260/3-8263/3, 8310/3, 8323/3, 8380/3, 8480/3, 8481/3, 8490/3, 8500/3, 8510/3, 8550/3, 8560/3, 8572/3, 8574/3, 8576/3
Cervical cancer	8051/3, 8052/3, 8070/3-8074/3, 8076/3, 8082/3-8084/3, 8140/3, 8144/3, 8200/3, 8210/3,8255/3, 8260/3-8263/3, 8310/3, 8323/3, 8380/3, 8382/3, 8384/3, 8441/3, 8460/3, 8480/3-8482/3, 8490/3, 8560/3, 8570/3, 8574/3
Differentiated thyroid cancer	8050/3, 8060/3, 8330/3-8332/3, 8335/3, 8340/3-8344/3, 8504/3, 8507/3

Table S2. The cancer-specific characteristics of five different cancers.

Variables	Subgroup	No. (%) of patients or Median (range)				
		**Lung (n=12,617)**	**Breast (n=166,051)**	**Thyroid (n=31,096)**	**Colorectal (n=23,550)**	**Cervical (n=8,347)**
Histology	Adenocarcinoma	8722(69.1)	/	/	23550(100.0)	2,684(32.2)
	Squamous	3895(30.9)	/	/	/	5,360(64.2)
	Adenosquamous	/	/	/	/	302(3.6)
	IDC	/	141730(85.4)	/	/	/
	ILC	/	14673(8.8)	/	/	/
	IDLC	/	9649(5.8)	/	/	/
	PTC	/	/	19,544(62.9)	/	/
	FTC	/	/	10,241(32.9)	/	/
	PTCfv	/	/	1,311(4.2)	/	/
Primary site	Upper lobe	7490(59.4)	/	/	/	/
	Mid lobe	602(4.8)	/	/	/	/
	Lower lobe	4181(33.1)	/	/	/	/
	Nipple and central	/	7,060(4.2)	/	/	/
	Inner	/	32,335(19.5)	/	/	/
	Outer	/	72,091(43.4)	/	/	/
	Right	/	/	/	10632(45.1)	/
	Left	/	/	/	6646(28.2)	/
	Rectum	/	/	/	5988(25.5)	/
	Other[a]	344(2.7)	54,565(32.9)	31,096(100.0)	284(1.2)	8,346(100.0)
Molecular subtype	HR+/HER2+	/	17,699(10.7)	/	/	/
	HR+/HER2-	/	12,4107(74.7)	/	/	/
	HR-/HER2+	/	6,888(4.1)	/	/	/
	HR-/HER2-	/	17,357(10.5)	/	/	/

Note: Other[a]: Not listed the detailed location of the primary tumor site;

Abbreviation: IDC: infiltrating ductal carcinoma; ILC: infiltrating lobular carcinoma; IDLC: infiltrating ductal mixed lobular carcinoma; PTC: papillary thyroid carcinoma; FTC: follicular thyroid carcinoma PTCfv: papillary thyroid carcinoma, follicular variate; HR: hormone receptor, HER-2: human epidermal growth factor receptor-2.

Table S3-1. Univariate and Multivariable Cox analyses of the association of treatment delays intervals with overall survival (treatment delays was calculated as continuous variable).

Cancer	Model 1		Model 2[a]	
	HR[b] (95%CI)	*P*	HR (95%CI)	*P*
**NSCLC**				
**Stage I**	1.20(1.17-1.23)	**<0.001**	1.04(1.01-1.06)	**0.010**
**Stage II**	1.10(1.06-1.14)	**<0.001**	1.00(0.96-1.04)	0.983
**IBC**				
**Stage I**	1.02(0.99-1.05)	0.150	1.03(1.00-1.06)	**0.029**
**Stage II**	1.00(0.98-1.03)	0.771	1.01(0.99-1.03)	0.377
**DTC**				
**Stage I**	0.76(0.68-0.87)	**<0.001**	0.94(0.83-1.06)	0.296
**Stage II**	0.71(0.52-0.98)	**0.038**	0.70(0.50-0.97)	**0.035**
**Colorectal cancer**				
**Stage I**	1.07(1.01-1.12)	**0.013**	0.97(0.92-1.03)	0.358
**Stage II**	0.95(0.91-0.99)	**0.017**	0.89(0.85-0.93)	**<0.001**
**Cervical cancer[c]**				
**Stage I**	1.16(1.09-1.24)	**<0.001**	0.95(0.89-1.03)	0.217
**Stage II**	1.02(0.95-1.10)	0.529	0.99(0.91-1.07)	0.833

Note: Model 2[a] was adjusted by the age, race, tumor location (exception of DTC, cervical cancer), differentiated grade; histology (exception of colorectal cancer), T stage, N stage (exception of NSCLC in stage I, DTC in stage II, colorectal cancer), surgery (exception of DTC), radiotherapy, chemotherapy (exception of DTC), marital status, income, and molecular subtype (only for breast cancer).

HR[b]: compared with immediate treatment initiation; Cervical cancer[c]: FIGO stage.

Abbreviation: HR: Hazard ratio, CI: confidence interval; NSCLC: non-small cell lung cancer, IBC: infiltrating breast cancer, DTC: differentiated thyroid carcinoma.

Bold values indicate statistical significance ($p<0.05$)

Table S3-2. Univariate and Multivariable Cox analyses of the association of treatment delays intervals with cancer-specific survival (treatment delays was calculated as continuous variable).

Cancer	Model 1		Model 2[a]	
	HR[b] (95%CI)	*P*	HR (95%CI)	*P*
**NSCLC**				
**Stage I**	1.20(1.16-1.24)	**<0.001**	1.02(0.98-1.06)	0.286
**Stage II**	1.06(1.02-1.11)	**0.002**	0.97(0.93-1.01)	0.144
**IBC**				
**Stage I**	0.97(0.91-1.02)	0.217	0.97(0.92-1.03)	0.321
**Stage II**	0.97(0.94-1.00)	**0.029**	0.99(0.97-1.02)	0.747
**DTC**				
**Stage I**	1.12(0.69-1.82)	0.649	1.04(0.61-1.77)	0.877
**Stage II**	0.13(0.00-10.92)	0.367	0.11(0.00-5.93)	0.277
**Colorectal cancer**				
**Stage I**	1.25(1.14-1.36)	**<0.001**	0.99(0.89-1.09)	0.795

**Stage II**	0.94(0.89-1.00)	**0.044**	0.83(0.78-0.88)	**<0.001**
**Cervical cancer[c]**				
**Stage I**	1.23(1.14-1.33)	**<0.001**	1.03(0.94-1.13)	0.480
**Stage II**	1.04(0.95-1.14)	0.380	1.02(0.93-1.12)	0.716

Note: Model 2[a] was adjusted by the age, race, tumor location (exception of DTC, cervical cancer), differentiated grade; histology (exception of colorectal cancer), T stage, N stage (exception of NSCLC in stage I, DTC in stage II, colorectal cancer), surgery (exception of DTC), radiotherapy, chemotherapy (exception of DTC), marital status, income, and molecular subtype (only for breast cancer).

HR[b]: compared with immediate treatment initiation; Cervical cancer[c]: FIGO stage.

Abbreviation: HR: Hazard ratio, CI: confidence interval; NSCLC: non-small cell lung cancer, IBC: infiltrating breast cancer, DTC: differentiated thyroid carcinoma.

Bold values indicate statistical significance ($p<0.05$)

Table S4-1. Multivariable analyses of the association of treatment delays intervals with overall survival in NSCLC (stage I).

Variables	Subgroup*	Hazard ratio (95%CI)	*P*
**Treatment delays intervals**	Immediate	Reference	
	Intermediate	1.04(0.95-1.13)	0.349
	Long	1.11(1.01-1.23)	**0.044**
**Race**	White	Reference	
	Black	0.90(0.80-1.01)	0.088
	other[a]	0.69(0.59-0.80)	**<0.001**
**Location**	Upper lobe	Reference	
	Mid lobe	0.98(0.84-1.15)	0.809
	Lower lobe	1.08(1.01-1.16)	**0.030**
	Other[b]	1.10(0.89-1.34)	0.373
**Grade**	I	Reference	
	II	1.68(1.51-1.87)	**<0.001**
	III/IV	2.00(1.77-2.23)	**<0.001**
	Unknown	1.61(1.41-1.83)	**<0.001**
**Histology**	Adenocarcinoma	Reference	
	Squamous	1.32(1.23-1.41)	**<0.001**
**T stage**	T1	Reference	
	T2	1.32(1.23-1.41)	**<0.001**
**Surgery**	Not performed	Reference	
	Performed	0.48(0.40-0.59)	**<0.001**
**Radiotherapy**	Not performed	Reference	
	Performed	1.32(1.10-1.59)	**0.003**
**Chemotherapy**	Not performed	Reference	
	Performed	1.15(1.03-1.28)	**0.014**
**Age**	/	1.03(1.03-1.03)	**<0.001**
**Marital status**	Married	Reference	
	Single	1.23(1.11-1.37)	**<0.001**
	Other[c]	1.19(1.11-1.28)	**<0.001**
	Unknown	1.02(0.87-1.19)	0.829
**Income**	< 35K	Reference	
	35K-<55K	0.92(0.76-1.11)	0.385
	55k-<75k	0.85(0.70-1.03)	0.088
	>75k	0.73(0.60-0.89)	**0.002**

Note: *subgroup: immediate: < 1 month, intermediate delayed: ≥1 and ≤2 months, and long delayed: ≥ 3 months; other[a]: defined as the Asian/Pacific Islander and American Indian/Alaska Native; Other[b]: Not listed the detailed location of the primary tumor site; Other[c]: Divorced, separated, windowed, Unmarried or Domestic Partner.

Abbreviation: CI: confidence interval; NSCLC: non-small cell lung cancer.

Bold values indicate statistical significance (p<0.05)

Table S4-2. Multivariable analyses of the association of treatment delays intervals with overall survival

in NSCLC (stage II).

Variables	Subgroup*	Hazard ratio (95%CI)	*P*
**Treatment delays intervals**	Immediate	Reference	
	Intermediate	0.97(0.87-1.09)	0.699
	Long	1.00(0.86-1.16)	0.995
**Race**	White	Reference	
	Black	0.92(0.79-1.07)	0.289
	other[a]	0.88(0.73-1.05)	0.162
**Location**	Upper lobe	Reference	
	Mid lobe	1.13(0.91-1.40)	0.270
	Lower lobe	1.11(1.01-1.22)	**0.032**
	Other[b]	1.06(0.87-1.29)	0.558
**Grade**	I	Reference	
	II	1.23(1.03-1.48)	**0.023**
	III/IV	1.44(1.20-1.73)	**<0.001**
	Unknown	1.18(0.96-1.45)	0.109
**Histology**	Adenocarcinoma	Reference	
	Squamous	1.11(1.01-1.22)	**0.027**
**T stage**	T1	Reference	
	T2	0.95(0.82-1.10)	0.482
	T3	0.92(0.76-1.11)	0.363
**N stage**	N0	Reference	
	N1	1.06(0.92-1.21)	0.421
**Surgery**	Not performed	Reference	
	Performed	0.42(0.36-0.49)	**<0.001**
**Radiotherapy**	Not performed	Reference	
	Performed	1.14(0.99-1.31)	0.077
**Chemotherapy**	Not performed	Reference	
	Performed	0.86(0.78-0.94)	**0.002**
**Age**	/	1.02(1.01-1.03)	**<0.001**
**Marital status**	Married	Reference	
	Single	1.09(0.94-1.26)	0.262
	Other[c]	1.04(0.94-1.14)	0.464
	Unknown	0.99(0.79-1.23)	0.899
**Income**	< 35K	Reference	
	35K-<55K	0.70(0.56-0.89)	**0.003**
	55k-<75k	0.64(0.51-0.80)	**<0.001**
	>75k	0.62(0.49-0.79)	**<0.001**

Note: *subgroup: immediate: < 1 month, intermediate delayed: ≥1 and ≤2 months, and long delayed: ≥ 3 months; other[a]: defined as the Asian/Pacific Islander and American Indian/Alaska Native; Other[b]: Not listed the detailed location of the primary tumor site; Other[c]: Divorced, separated, windowed, Unmarried or Domestic Partner.

Table S5-1. Multivariable analyses of the association of treatment delays intervals with overall survival in infiltrating breast cancer (stage I).

Variables	Subgroup*	Hazard ratio (95%CI)	*P*
**Treatment delays intervals**	Immediate	Reference	
	Intermediate	0.92(0.87-0.97)	**0.003**
	Long	1.23(1.11-1.37)	**<0.001**
**Race**	White	Reference	
	Black	1.22(1.13-1.32)	**<0.001**
	other[a]	0.72(0.65-0.80)	**<0.001**
**Location**	Nipple and central	Reference	
	Inner	0.97(0.86-1.10)	0.662
	Outer	0.96(0.86-1.08)	0.531
	Other[b]	0.98(0.87-1.11)	0.801
**Grade**	I	Reference	
	II	1.15(1.09-1.22)	**<0.001**
	III/IV	1.61(1.50-1.74)	**<0.001**
	Unknown	1.07(0.91-1.26)	0.391
**Histology**	IDC	Reference	
	ILC	0.93(0.85-1.02)	0.213
	IDLC	0.95(0.85-1.06)	0.327
**N stage**	N0	Reference	
	N1	1.44(1.31-1.60)	**<0.001**
**Surgery**	Not performed	Reference	
	Performed	0.23(0.18-0.31)	**<0.001**
**Radiotherapy**	Not performed	Reference	
	Performed	0.62(0.59-0.65)	**<0.001**
**Chemotherapy**	Not performed	Reference	
	Performed	1.06(0.99-1.14)	0.109
**Age**	/	1.08(1.07-1.08)	**<0.001**
**Marital status**	Married	Reference	
	Single	1.46(1.35-1.58)	**<0.001**
	Other[c]	1.54(1.46-1.62)	**<0.001**
	Unknown	1.27(1.14-1.43)	**<0.001**
**Income**	< 35K	Reference	
	35K-<55K	0.88(0.75-1.04)	0.142
	55k-<75k	0.70(0.60-0.83)	**<0.001**
	>75k	0.60(0.51-0.71)	**<0.001**
**Subtype**	HR+/HER2+	Reference	
	HR+/HER2-	1.08(0.98-1.20)	0.117
	HR-/HER2+	1.06(0.90-1.24)	0.468
	HR-/HER2-	1.50(1.34-1.67)	**<0.001**

Note: *subgroup: immediate: < 1 month, intermediate delayed: ≥1 and ≤2 months, and long delayed: ≥ 3 months; other[a]: defined as the Asian/Pacific Islander and American Indian/Alaska Native; Other[b]: Not listed the detailed location of the primary tumor site; Other[c]: Divorced, separated, windowed,

Unmarried or Domestic Partner.
Abbreviation: CI: confidence interval; HR: hormone receptor, HER-2: human epidermal growth factor receptor-2.
Bold values indicate statistical significance (*p*<0.05)

Table S5-2. Multivariable analyses of the association of treatment delays intervals with overall survival in infiltrating breast cancer (stage II).

Variables	Subgroup*	Hazard ratio (95%CI)	*P*
**Treatment delays intervals**	Immediate	Reference	
	Intermediate	0.93(0.88-0.97)	**0.002**
	Long	1.09(0.99-1.19)	0.059
**Race**	White	Reference	
	Black	1.22(1.15-)	**<0.001**
	other[a]	0.77(0.71-0.84)	**<0.001**
**Location**	Nipple and central	Reference	
	Inner	1.06(0.96-1.17)	0.236
	Outer	0.89(0.82-0.98)	0.018
	Other[b]	1.00(0.91-1.10)	0.978
**Grade**	I	Reference	
	II	1.43(1.32-1.55)	**<0.001**
	III/IV	2.16(1.98-2.35)	**<0.001**
	Unknown	1.87(1.62-2.15)	**<0.001**
**Histology**	IDC	Reference	
	ILC	0.83(0.77-0.89)	**<0.001**
	IDLC	0.83(0.76-0.92)	**<0.001**
**T stage**	T1	Reference	
	T2	1.76(1.65-1.89)	**<0.001**
	T3	2.76(2.47-3.09)	**<0.001**
**N stage**	N0	Reference	
	N1	1.70(1.62-1.78)	**<0.001**
**Surgery**	Not performed	Reference	
	Performed	0.47(0.42-0.52)	**<0.001**
**Radiotherapy**	Not performed	Reference	
	Performed	0.78(0.74-0.81)	**<0.001**
**Chemotherapy**	Not performed	Reference	
	Performed	0.72(0.69-0.76)	**<0.001**
**Age**	/	1.04(1.04-1.04)	**<0.001**
**Marital status**	Married	Reference	
	Single	1.34(1.27-1.43)	**<0.001**
	Other[c]	1.34(1.28-1.41)	**<0.001**
	Unknown	1.27(1.15-1.39)	**<0.001**
**Income**	< 35K	Reference	
	35K-<55K	0.79(0.69-0.90)	**<0.001**
	55k-<75k	0.65(0.57-0.74)	**<0.001**

	>75k	0.58(0.51-0.66)	**<0.001**
**Subtype**	HR+/HER2+	Reference	
	HR+/HER2-	1.20(1.11-1.29)	**<0.001**
	HR-/HER2+	1.16(1.04-1.29)	**<0.001**
	HR-/HER2-	2.20(2.02-2.38)	**<0.001**

Note: *subgroup: immediate: < 1 month, intermediate delayed: ≥1 and ≤2 months, and long delayed: ≥ 3 months; other[a]: defined as the Asian/Pacific Islander and American Indian/Alaska Native; Other[b]: Not listed the detailed location of the primary tumor site; Other[c]: Divorced, separated, windowed, Unmarried or Domestic Partner.

Abbreviation: CI: confidence interval; HR: hormone receptor, HER-2: human epidermal growth factor receptor-2.

Bold values indicate statistical significance (p<0.05)

Table S6-1. Multivariable analyses of the association of treatment delays intervals with overall survival in DTC (stage I).

Variables	Subgroup*	Hazard ratio (95%CI)	*P*
**Treatment delays intervals**	Immediate	Reference	
	Intermediate	0.70(0.53-0.93)	**0.012**
	Long	1.09(0.70-1.70)	0.711
**Race**	White	Reference	
	Black	1.71(1.30-2.25)	**<0.001**
	other[a]	0.44(0.26-0.73)	**0.001**
**Grade**	I	Reference	
	II	0.60(0.30-1.19)	0.145
	III/IV	5.00(2.14-11.66)	**<0.001**
	Unknown	0.89(0.71-1.12)	0.326
**Histology**	PTC	Reference	
	FTC	1.11(0.91-1.36)	0.288
	PTCfv	1.95(1.21-3.13)	**0.006**
**T stage**	T1a	Reference	
	T1b	1.04(0.83-1.32)	0.710
	T2	0.99(0.50-1.96)	0.977
	T3	1.54(0.82-2.89)	0.179
	T4	8.59(3.61-20.45)	**<0.001**
**N stage**	N0	Reference	
	N1a	1.44(0.76-2.74)	0.265
	N1b	1.03(0.39-2.71)	0.953
**Radiotherapy**	Not performed	Reference	
	Performed	0.69(0.53-0.89)	**0.004**
**Age**	/	1.09(1.08-1.10)	**<0.001**
**Marital status**	Married	Reference	
	Single	1.81(1.39-2.35)	**<0.001**
	Other[c]	1.59(1.26-2.00)	**<0.001**
	Unknown	1.02(0.65-1.59)	0.946
**Income**	< 35K	Reference	
	35K-<55K	1.02(0.54-1.94)	0.953
	55k-<75k	0.76(0.40-1.45)	0.412
	>75k	0.68(0.35-1.32)	0.256

Note: *subgroup: immediate: < 1 month, intermediate delayed: ≥1 and ≤2 months, and long delayed: ≥ 3 months; other[a]: defined as the Asian/Pacific Islander and American Indian/Alaska Native; Other[b]: Not listed the detailed location of the primary tumor site; Other[c]: Divorced, separated, windowed, Unmarried or Domestic Partner.

Abbreviation: CI: confidence interval; DTC: differentiated thyroid carcinoma.

Bold values indicate statistical significance (p<0.05)

Table S6-2. Multivariable analyses of the association of treatment delays intervals with overall survival in DTC (stage II).

Variables	Subgroup*	Hazard ratio (95%CI)	*P*
**Treatment delays intervals**	Immediate	Reference	
	Intermediate	0.73(0.41-1.31)	0.300
	Long	0.22(0.03-1.61)	0.138
**Race**	White	Reference	
	Black	1.28(0.62-2.67)	0.503
	other[a]	1.45(0.65-3.26)	0.366
**Grade**	I	Reference	
	II	1.38(0.46-4.21)	0.564
	III/IV	3.47(0.44-27.16)	0.236
	Unknown	0.86(0.49-1.51)	0.608
**Histology**	PTC	Reference	
	FTC	1.73(1.00-2.99)	0.050
	PTCfv	1.46(0.74-2.87)	0.278
**Radiotherapy**	Not performed	Reference	
	Performed	0.42(0.26-0.68)	**<0.001**
**Age**	/	1.10(1.07-1.13)	**<0.001**
**Marital status**	Married	Reference	
	Single	2.58(1.31-5.07)	**0.006**
	Other[c]	1.71(1.01-2.89)	**0.047**
	Unknown	0.40(0.05-3.00)	0.375
**Income**	< 35K	Reference	
	35K-<55K	0.63(0.24-1.67)	0.359
	55k-<75k	0.26(0.10-0.71)	**0.008**
	>75k	0.30(0.11-0.84)	**0.022**

Note: *subgroup: immediate: < 1 month, intermediate delayed: ≥1 and ≤2 months, and long delayed: ≥ 3 months; other[a]: defined as the Asian/Pacific Islander and American Indian/Alaska Native; Other[b]: Not listed the detailed location of the primary tumor site; Other[c]: Divorced, separated, windowed, Unmarried or Domestic Partner.

Abbreviation: CI: confidence interval; DTC: differentiated thyroid carcinoma.

Bold values indicate statistical significance (*p*<0.05)

Table S7-1. Multivariable analyses of the association of treatment delays intervals with overall survival in colorectal cancer (stage I).

Variables	Subgroup*	Hazard ratio (95%CI)	*P*
**Treatment delays intervals**	Immediate	Reference	
	Intermediate	0.83(0.75-0.92)	**<0.001**
	Long	1.21(0.97-1.52)	0.090
**Race**	White	Reference	
	Black	1.17(1.02-1.34)	**0.027**
	other[a]	0.78(0.65-0.94)	**0.008**
**Location**	Right	Reference	
	Left	1.03(0.92-1.15)	0610
	Rectum	1.06(0.94-1.20)	0.324
	Other[b]	1.50(1.00-2.26)	**0.049**
**Grade**	I	Reference	
	II	1.02(0.89-1.16)	0.789
	III/IV	1.15(0.95-1.40)	0.161
	Unknown	0.93(0.75-1.15)	0.498
**T stage**	T1	Reference	
	T2	1.16(1.06-1.28)	**0.001**
**Surgery**	Not performed	Reference	
	Performed	0.29(0.22-0.39)	**<0.001**
**Radiotherapy**	Not performed	Reference	
	Performed	0.94(0.67-1.29)	0.711
**Chemotherapy**	Not performed	Reference	
	Performed	1.58(1.17-2.12)	**0.003**
**Age**	/	1.08(1.07-1.08)	**<0.001**
**Marital status**	Married	Reference	
	Single	1.62(1.40-1.87)	**<0.001**
	Other[c]	1.38(1.24-1.53)	**<0.001**
	Unknown	1.14(0.95-1.37)	0.168
**Income**	< 35K	Reference	
	35K-<55K	0.93(0.70-1.23)	0.613
	55k-<75k	0.76(0.68-1.00)	0.056
	>75k	0.64(0.48-0.85)	**0.002**

Note: *subgroup: immediate: < 1 month, intermediate delayed: ≥1 and ≤2 months, and long delayed: ≥ 3 months; other[a]: defined as the Asian/Pacific Islander and American Indian/Alaska Native; Other[b]: Not listed the detailed location of the primary tumor site; Other[c]: Divorced, separated, windowed, Unmarried or Domestic Partner.

Abbreviation: CI: confidence interval.

Bold values indicate statistical significance (p<0.05)

Table S7-2. Multivariable analyses of the association of treatment delays intervals with overall survival in colorectal cancer (stage II).

Variables	Subgroup*	Hazard ratio (95%CI)	*P*

**Treatment delays intervals**	Immediate	Reference	
	Intermediate	0.70(0.65-0.75)	**<0.001**
	Long	0.87(0.72-1.04)	0.120
**Race**	White	Reference	
	Black	1.18(1.07-1.31)	**0.002**
	other[a]	0.82(0.72-0.93)	**0.003**
**Location**	Right	Reference	
	Left	1.17(1.08-1.27)	**<0.001**
	Rectum	1.36(1.21-1.53)	**<0.001**
	Other[b]	1.18(0.92-1.51)	**<0.001**
**Grade**	I	Reference	0.203
	II	1.08(0.94-1.23)	0.290
	III/IV	1.21(1.04-1.41)	**0.012**
	Unknown	1.19(0.95-1.48)	0.129
**T stage**	T3	Reference	
	T4	2.19(2.02-2.37)	**<0.001**
**Surgery**	Not performed	Reference	
	performed	0.29(0.24-0.34)	**<0.001**
**Radiotherapy**	Not performed	Reference	
	performed	1.08(0.94-1.26)	0.278
**Chemotherapy**	Not performed	Reference	
	performed	0.82(0.74-0.91)	**<0.001**
**Age**	/	1.05(1.05-1.06)	**<0.001**
**Marital status**	Married	Reference	
	Single	1.48(1.33-1.64)	**<0.001**
	Other[c]	1.33(1.23-1.44)	**<0.001**
	Unknown	1.19(1.02-1.38)	**0.029**
**Income**	< 35K	Reference	
	35K-<55K	0.83(0.68-1.01)	0.066
	55k-<75k	0.73(0.60-0.89)	**0.002**
	>75k	0.68(0.55-0.83)	**<0.001**

Note: *subgroup: immediate: < 1 month, intermediate delayed: ≥1 and ≤2 months, and long delayed: ≥ 3 months; other[a]: defined as the Asian/Pacific Islander and American Indian/Alaska Native; Other[b]: Not listed the detailed location of the primary tumor site; Other[c]: Divorced, separated, windowed, Unmarried or Domestic Partner.

Abbreviation: CI: confidence interval.

Bold values indicate statistical significance (*p*<0.05)

Table S8-1. Multivariable analyses of the association of treatment delays intervals with overall survival in cervical cancer (stage I).

Variables	Subgroup*	Hazard ratio (95%CI)	*P*
**Treatment delays intervals**	Immediate	Reference	
	Intermediate	1.08(0.90-1.30)	0.421
	Long	0.84(0.63-1.14)	0.282
**Race**	White	Reference	
	Black	1.62(1.30-2.02)	**<0.001**
	other[a]	0.91(0.68-1.22)	0.546
**Grade**	I	Reference	
	II	1.34(0.99-1.81)	0.061
	III/IV	2.29(1.69-3.10)	**<0.001**
	Unknown	1.36(0.98-1.88)	**0.064**
**Histology**	Squamous	Reference	
	Adenocarcinoma	1.05(0.86-1.28)	0.621
	Adenosquamous	1.25(0.85-1.83)	0.255
**T stage**	T1	Reference	
	T2	2.03(0.94-4.40)	0.071
**Surgery**	Not performed	Reference	
	Performed	0.47(0.37-0.59)	**<0.001**
**Radiotherapy**	Not performed	Reference	
	Performed	2.34(1.85-2.97)	**<0.001**
**Chemotherapy**	Not performed	Reference	
	Performed	0.96(0.77-1.21)	0.740
**Age**	/	1.03(1.02-1.04)	**<0.001**
**Marital status**	Married	Reference	
	Single	1.16(0.95-1.43)	0.147
	Other[c]	1.30(1.06-1.61)	**0.013**
	Unknown	1.16(0.82-1.64)	0.402
**Income**	< 35K	Reference	
	35K-<55K	1.08(0.62-1.87)	0.787
	55k-<75k	1.10(0.64-1.90)	0.726
	>75k	0.79(0.45-1.39)	0.413

Note: *subgroup: immediate: < 1 month, intermediate delayed: ≥1 and ≤2 months, and long delayed: ≥ 3 months; other[a]: defined as the Asian/Pacific Islander and American Indian/Alaska Native; Other[b]: Not listed the detailed location of the primary tumor site; Other[c]: Divorced, separated, windowed, Unmarried or Domestic Partner.

Abbreviation: CI: confidence interval.

Bold values indicate statistical significance ($p<0.05$)

Table S8-2. Multivariable analyses of the association of treatment delays intervals with overall survival in cervical cancer (stage II).

Variables	Subgroup*	Hazard ratio (95%CI)	*P*
**Treatment delays intervals**	Immediate	Reference	

	Intermediate	0.92(0.74-1.14)	0.436
	Long	1.01(0.75-1.37)	0.949
**Race**	White	Reference	
	Black	1.19(0.95-1.49)	0.139
	other[a]	0.99(0.76-1.28)	0.938
**Grade**	I	Reference	
	II	1.46(0.95-2.25)	0.087
	III/IV	1.54(0.99-2.38)	0.054
	Unknown	1.54(0.99-2.38)	0.057
**Histology**	Squamous	Reference	
	Adenocarcinoma	1.23(0.99-1.53)	0.058
	Adenosquamous	1.18(0.80-1.74)	0.408
**Surgery**	Not performed	Reference	
	performed	0.77(0.62-0.95)	**0.017**
**Radiotherapy**	Not performed	Reference	
	performed	0.61(0.42-0.89)	**0.011**
**Chemotherapy**	Not performed	Reference	
	performed	0.99(0.76-1.32)	0.964
**Age**	/	1.01(1.00-1.02)	**<0.001**
**Marital status**	Married	Reference	
	Single	1.23(0.99-1.51)	0.058
	Other[c]	1.22(0.98-1.52)	0.074
	Unknown	1.23(0.82-1.85)	0.315
**Income**	< 35K	Reference	
	35K-<55K	0.86(0.52-1.42)	0.560
	55k-<75k	0.82(0.50-1.35)	0.447
	>75k	0.73(0.43-1.22)	0.227

Note: *subgroup: immediate: < 1 month, intermediate delayed: ≥1 and ≤2 months, and long delayed: ≥ 3 months; other[a]: defined as the Asian/Pacific Islander and American Indian/Alaska Native; Other[b]: Not listed the detailed location of the primary tumor site; Other[c]: Divorced, separated, windowed, Unmarried or Domestic Partner.

Abbreviation: CI: confidence interval.

Bold values indicate statistical significance (p<0.05)
